# Surgeons have hesitated early cholecystectomy because of cardiovascular comorbidities during adoption of guidelines

**DOI:** 10.1038/s41598-021-04479-y

**Published:** 2022-01-11

**Authors:** Ichiro Onishi, Masato Kayahara, Takahisa Yamaguchi, Yukari Yamaguchi, Akihiko Morita, Nariatsu Sato, Yoshiyuki Kurosaka, Shigeru Takegawa

**Affiliations:** Department of Surgery, National Hospital Organization Kanazawa Medical Center, 1-1 Shimoishibiki, Kanazawa, 920-8650 Japan

**Keywords:** Health care, Medical research

## Abstract

The introduction of the guidelines has resulted in an increase of laparoscopic surgeries performed, but the rate of early surgery was still low. Here, the initial effect of the introduction of the guideline was confirmed in single center, and factors disturbing early cholecystectomy were analyzed. This study included 141 patients who were treated for acute cholecystitis from January 2010 to October 2014 at Kanazawa Medical Center. Each patient was assigned into a group according to when they received treatment. Patients in Group A were treated before the Tokyo Guidelines were introduced (n = 48 cases), those in Group B were treated after the introduction of the guidelines (93 cases). After the introduction of the guidelines, early laparoscopic cholecystectomy was significantly increased (P < 0.001), however, the rate of early operations was still 38.7% only. There are many cases with cardiovascular disease in delayed group, the prevalence had reached 50% as compared with early group of 24% (P < 0.01). Approximately 25% of patients continued antiplatelet or anticoagulant therapy. In the early days of guidelines introduction, the factor which most disturbed early surgery was the coexistence of cardiovascular disease. These contents could be described in the next revision of the guidelines.

## Introduction

Until the Tokyo Guidelines were published, there have been no evidence-based criteria for the diagnosis, severity assessment, or treatment of acute cholecystitis^1–3^. Many reports on acute cholecystitis use different standards and comparisons have therefore been difficult^4,5^. Laparoscopic cholecystectomy has been found to be superior to open cholecystectomy for the treatment of acute cholecystitis because of shorter duration of postoperative hospital stay, quicker recuperation, and earlier return to work^1–5^. Therefore, according to the guidelines, the principal management of acute cholecystitis is early cholecystectomy.

The introduction of the guidelines has resulted in an increase in the number of laparoscopic surgeries performed each year, and then the perioperative hospitalization period has shortened, but the rate of early surgery was still low^6,7^. Because early laparoscopic cholecystectomy for acute cholecystitis has not been routine in most of Japanese general hospital since the publication of the guidelines because the timing and approach of surgical management of patients with acute cholecystitis remain a matter of controversy^6–10^. In addition, it became difficult to judge by the spread of covid-19 infection, despite the need to secure a hospital bed.

Here, the initial effect of the introduction of the guideline was confirmed in Japanese single center, and factors preventing early cholecystectomy were analyzed.

## Methods

### Ethics statements

All methods were conducted in accordance with relevant guidelines and regulations. This study was approved by the Institutional Review Boards of Kanazawa Medical Center. Informed consent was obtained from all the participants.

### Patients

This study included 141 patients who were treated for acute cholecystitis from January 2010 to October 2014 at Kanazawa Medical Center (Table 1). At the time of admission, Ultrasound (US) showed morphological features of acute cholecystitis. Moreover, abdominal computed tomography (CT) and magnetic resonance cholangiopancreatograph**y** (MRCP) were performed, and the diagnosis of acute cholecystitis was confirmed.

**Table 1:** Patient characteristics.

Acute cholecystitis (141 cases)	A: Pre TG (48 cases)	B: Post TG (93 cases)	
**Age**	69.9 ± 13.5	65.9 ± 16.4	
< 75	29 (60.4%)	59 (63.4%)	NS
74<	19 (39.6%)	34 (36.6%)	
Male	20 (41.7%)	55 (59.1%)	NS
Female	28 (58.3%)	38 (40.9%)	
Grade I	36 (75%)	80 (86%)	NS
Grade II	12 (25%)	13 (14%)	
Comorbidities	34 (70.8%)	55 (59.1%)	NS
Cardiovascular	26 (54.2%)	37 (39.8%)	NS
Cerebrovascular	3 (6.3%)	4 (4.3%)	NS
Psychiatric	6 (12.5%)	18 (19.4%)	NS
Respiratory	4 (8.3%)	3 (3.2%)	NS

141 patients were examined divided into two groups A; before guideline introduced: 48 cases, B; after the introduction: 93 cases. No patient had Grade III disease. More than 50% of patients had comorbidities, especially cardiovascular diseases.

The following data were also collected such as serum creatinine level, international normalized ratio of prothrombin time (PT/INR), white blood cell count, platelet count, and albumin level etc.

Then the degree of severity of cholecystitis was categorized as Grade I (mild), Grade II (moderate), or Grade III (severe) based on the TG18 diagnostic criteria^1^. Based on the Tokyo guidelines diagnostic criteria, 116 (82.3%) and 25 (17.7%) patients were diagnosed with Grade I and II acute cholecystitis. No patient had Grade III disease.

More than 50% of patients had comorbidities, especially cardiovascular diseases. The patient characteristics in each group were essentially uniform (Table 1). All the patients received intravenous antibiotics upon admission, which was continued after surgery.

### Operative procedure and two groups comparison

Laparoscopic cholecystectomy was performed using the conventional four-port method with pneumoperitoneum installed via sub-umbilical mini-laparotomy and with the intraabdominal pressure kept at 8 mmHg. Each patient was assigned into a group according to when they received treatment. Patients in Group A were treated before the Tokyo Guidelines were introduced to our hospital (n = 48 cases), those in Group B were treated after the introduction of the guidelines (93 cases). The two groups were compared with respect to perioperative data, including the duration of surgery, conversion to open surgery, postoperative complications, and duration of postoperative hospital stay. These data were retrieved from surgical documentation sheets, surgeons’ notes, and discharge records. All surgeries in the present study were performed by experienced attending surgeons.

### Statistical analysis

Data are presented as the mean ± standard deviation (SD). Categorical data are presented as number (n) and percentage (%). Mann–Whitney U test was used for comparisons of the mean value between the two groups, statistical analysis for tables’ cells was performed using Fisher’s exact test at the 0.05 level of significance. Spearman Rank Order Correlation analysis is performed for preoperative period investigation. All statistical analyses were performed using SPSS for Windows, version 23.0 (SPSS, Chicago, IL, USA).

## Results

### Cholecystectomy

The operative details are provided in Table 2. Since the introduction of the Tokyo Guidelines, the number and proportion of laparoscopic cholecystectomy has increased compared with those of open cholecystectomy. After the introduction of the guidelines, laparoscopic cholecystectomy accounted for 81 (87.1%) of 93 cases. There were significant differences during the two groups regarding the type of surgery performed (P = 0.014), but the operation time did not differ significantly. We also compared the operation time according to surgical procedure, but no statistical significance was observed. Hospital stay was significantly shorter between groups A, and B (P < 0.001). It was inversely proportional to the number of laparoscopic surgeries performed. The timing of cholecystectomy is provided in Table 3.

**Table 2:** Procedure of cholecystectomy and hospital stay.

Acute cholecystitis (141 cases)	A: Pre TG (48 cases)	B: Post TG (93 cases)	
Laparoscopic	32 (66.7%)	81 (87.1%)	
Open	13 (27%)	10 (10.8%)	P = 0.014
(Convert)	3 (6.3%)	2 (2.1%)	
Operation time	127.7 ± 62.6	130.8 ± 44.3	NS
Laparoscopic	109.4 ± 60.2	129.7 ± 41.6	NS
Open	117.4 ± 64.9	130.7 ± 57.2	NS
(Convert)	189.5 ± 58.4	190 ± 24	NS
Hospital stay	22.7 ± 20.2	13.2 ± 14.5	P < 0.001

There were significant differences between the two groups regarding the type of surgery performed (P = 0.014). Hospital stay was significantly shorter in groups B, as compared with group A (P < 0.001).

**Table 3:** Timing of cholecystectomy.

Acute cholecystitis (141 cases)	A: Pre TG (48 cases)	B: Post TG (93 cases)	
**Laparoscopic**			
72 h	26 (54%)	48 (51%)	P < 0.001
≤ 72 h	1 (2.1%)	33 (35%)	
**Convert**			
72 h	8 (16%)	2 (2.2%)	NS
≤ 72 h	0	0	
**Laparotomy**			
72 h	9 (18%)	7 (7.5%)	NS
≤ 72 h	4 (8.3%)	3 (3.2%)	

After the introduction, early laparoscopic cholecystectomy was significantly increased (P < 0.001). However, the rate of early operations was still 38.7% only.

Only one early laparoscopic cholecystectomy had been performed, within 72 h from onset, before the introduction of the guidelines.

After the introduction, early laparoscopic cholecystectomy was significantly increased (P < 0.001). However, the rate of early operations was still 38.7% only.

### Preoperative period

To investigate the impact of the guidelines on the waiting time required for surgery, we investigated the duration from disease onset to surgery.

After the introduction of the Tokyo Guidelines, the time from onset to surgery was shortened (P = 0.017) as compared with that before. In addition, we have examined these periods in detail, then both primary care to admission and onset to admissions were shortened significantly.

But the time from admission to surgery did not change so much in each group (Table 4). Next, we examined the correlation between the two preoperative period to investigate the effect of each period on the time from onset to surgery.

**Table 4:** Preoperative period.

Acute cholecystitis (141 cases)	A: Pre TG (48 cases)	B: Post TG (93 cases)	
Onset to surgery	38.4 ± 53.3	24.8 ± 26.5	P = 0.017
Primary care to admission	14.1 ± 13.3	9.1 ± 12.6	P < 0.001
Onset to admission	13.9 ± 13.5	8.9 ± 11.9	P = 0.005
Admission to surgery	24.2 ± 52.1	15.9 ± 20.1	NS

After the introduction of the Tokyo Guidelines, the time from onset to surgery was shortened (p = 0.017) as compared with that before. But the time from admission to surgery did not change so much in each group.

**Figure 1:**
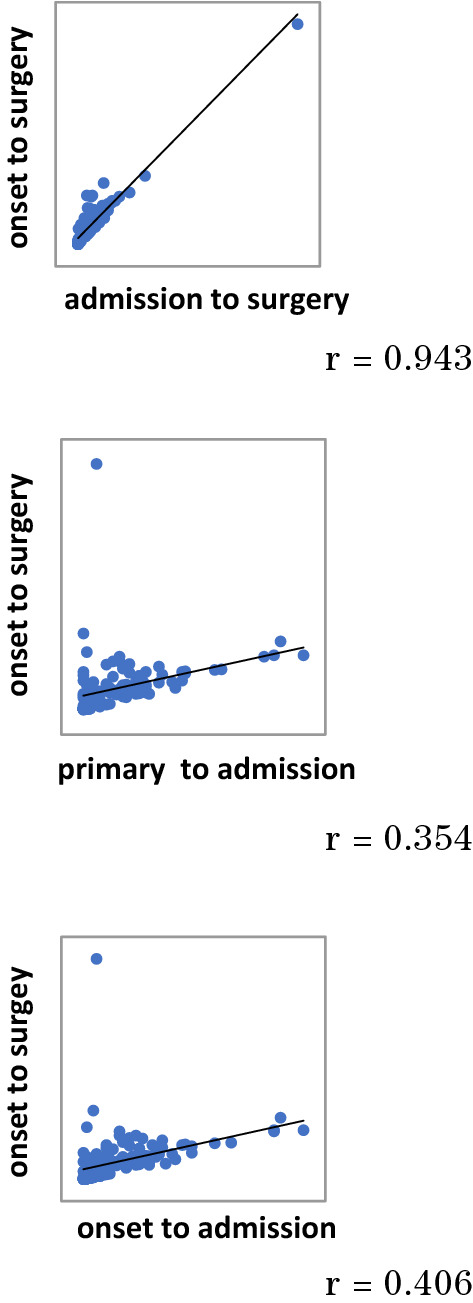
Correlation between preoperative periods. The most correlated period was the time from admission to surgery (r = 0.943), time from onset and primary care to admission had not significantly affected to the duration from onset to surgery.

The most correlated period was the time from admission to surgery (r = 0.943), time from onset and primary care to admission had not significantly affected to the duration from onset to surgery (Fig. 1). These changes are thought to have been brought about by prompt consultation from family physicians, indicating that the guidelines were widely recognized by primary care physicians, however, there are still little number of cases of early operation based on the appropriate judgment of surgeon, and this seems to have led to an extension of the extra waiting period.

### Laparoscopic cholecystectomy and comorbidities

As mentioned so far, after introducing guidelines early laparoscopic cholecystectomy increased little by little, however its ratio was not so high in the case of mild cholecystitis. Considering the relation of comorbid disease as the cause of delayed cholecystectomy, we checked the comorbidity diseases between early cholecystectomy and interval cholecystectomy (Table 5).

**Table 5:** Laparoscopic cholecystectomy and comorbidities.

Post TG laparoscopic (81 cases)	Laparoscopic 72 h < (48 cases)	Laparoscopic ≤ 72 h (33 cases)	
Comorbidities	31 (64%)	17 (48%)	NS
Cardiovascular	24 (50%)	8 (24%)	P = 0.0087
Cerebrovascular	1 (2%)	0 (0%)	NS
Psychiatric	9 (1.9%)	6 (18%)	NS
Respiratory	0 (0%)	2 (6%)	NS

There are many cases with cardiovascular disease in delayed group, the prevalence had reached 50% as compared with early group of 24% (P =0.0087).

As a result, there are many cases with cardiovascular disease in delayed group, the prevalence had reached 50% as compared with early group of 24% (P =0.0087). Approximately 25% of patients continued antiplatelet or anticoagulant therapy, therefore surgeons might have avoided early surgery (Table 6). Then the discontinuation of these drugs was forced for extra admission for the heparinization before the operation. Surgeons feared cardiovascular disease and the effects of antiplatelet or anticoagulant therapy too much, then they could not get to the early surgery.

**Table 6:** Cardiovascular disease and antithrombotic therapy.

Post TG laparoscopic (81 cases)	Laparoscopic 72 h < (48 cases)	Laparoscopic ≤ 72 h (33 cases)
HT	21 (43%)	7 (21%)
OMI	1 (2%)	0
DVT	1 (2%)	0
AAA	2 (4.1%)	0
Angina	2 (4.1%)	0
ASO	1 (2%)	0
Af	0	1 (3%)
Antiplatelet therapy	12 (25%)	1 (3%)
Anticoagulation therapy	0	1 (3%)

Approximately 25% of patients continued antiplatelet or anticoagulant therapy.

### Complications

The main concern in the introduction of the guidelines is an increase in complications of laparoscopic cholecystectomy. In this case series the number of complications was low, but little bit increase was revealed during the application period (Table 7). No serious complications occurred with laparoscopic surgery, but small bile leakage was observed in three patients, then both were cured by few days’ drainage only. In long-term follow-up after surgery, one patient needed re-operation and biliary reconstruction by bile duct stones centered on a clip and Mirizzi syndrome three years after the initial operation. In addition, this case was one of 4 cases of partial cholecystectomy.

**Table 7:** Complications.

Acute cholecystitis (141 cases)	A: Pre TG (48 cases)		B: Post TG (93 cases)	
	72 h	≤ 72 h	72 h	≤ 72 h
Bleeding	–	–	–	1 (1.1%)
Laparoscopic				1
Open				0
SSI	–	1 (2%)	0	–
Laparoscopic		0		
Open		1		
Bile leakage	–	–	2 (2.2%)	1 (1.1%)
Laparoscopic			2	0
Open			0	1
Residual stone	–	–	1 (1.1%)	1 (1.1%)
Laparoscopic			1	1
Open			0	0
Cholangitis	–	–	–	1 (1.1%)
Laparoscopic				1
Open				0
Pneumonia	–	–	–	1 (1.1%)
Laparoscopic				1
Open				0

The number of complications was low, but little bit increase was revealed during the application period. In long-term follow-up after surgery, one patient needed re-operation and biliary reconstruction by bile duct stones centered on a clip and Mirizzi syndrome three years after the initial operation. In addition, this case was one of 4 cases of partial cholecystectomy.

However, there was no increase of severity in comorbidity during the perioperative and long-term observation period after surgery.

## Discussion

After several open symposia and a survey of acute cholecystitis cases, a Japanese-language version of Evidence-Based Practice Guidelines for the Management of Acute Cholangitis and Cholecystitis has been developed and published in 2005. Then international experts discussed these in the Consensus Meeting, held on April 1–2, 2006, in Tokyo^11^, and they developed English-language version of International Guidelines as the Tokyo Guidelines. Before the Tokyo Guidelines were introduced, diagnostic and therapeutic strategies for acute cholecystitis had not yet been established. Sometimes, treatments were not consistent between doctors, and this led to inadequate treatment with poor results^12^.

Laparoscopic cholecystectomy for acute cholecystitis used to be associated with high complication and conversion rates and was not performed routinely as it was also known that the initial conservative treatment could be followed by interval elective surgery^5^.

Therefore, a flowchart for the management of acute cholecystitis according to severity is included in these guidelines^3^, to reduce the overall cost of treatment, routine early laparoscopic cholecystectomy was recommended^1,2^. In this case series, after the introduction of the Tokyo Guidelines, laparoscopic surgery rates for acute cholecystitis reached 87.1%. These changes led to shorter hospital stays, but no severe complications were observed during this period. The cost reduction is also a big advantage of laparoscopic cholecystectomy by shortening the treatment period^13^.

However, the introduction of the Tokyo Guidelines has not increased the number of early laparoscopic surgeries for acute cholecystitis as expected. After the introduction of the Tokyo Guidelines, the time from onset to surgery was shortened as compared with that before, then both primary care to admission and onset to admissions were shortened significantly. But the time from admission to surgery did not change so much in each group. The most correlated period was the time from admission to surgery, these changes are thought to have been brought from which the guidelines were widely recognized by primary care physicians, however, there are still little number of cases of early operation based on the appropriate judgment of surgeon, and this seems to have led to an extension of the extra waiting period.

Early laparoscopic surgeries could also contribute to shorter hospital stays and lower medical costs of hospital stays without complication. Indeed, there is no difference in complaints such as postoperative pain and nausea between early operation and elective operation^14^. In addition, early surgery is also effective even in the elderly, who has any kind of comorbidities^15,16^.

There are reports that comorbidities do not affect the outcome of early surgery in mild cholecystitis, moreover it may be introduced in severe cases^17^.

In patients with cardiovascular disease, gallstone cholecystitis is common from the viewpoint of metabolic syndrome and pharmacotherapy. Even in patients with severe cardiovascular disease, the removal of the waiting laparoscopic cholecystectomy with heparin bridge is the first choice^18^, but the safety in the case of early surgery has also been reported one after another in recent years. Around TG13, cases more than 96 h after onset and male were considered risk factors for conversion to laparotomy^19^. As the severity increases, postoperative complications increase relatively and become more costly. If the anatomy is not clear, the surgeon should not hesitate to convert to open cholecystectomy to prevent severe complications.

In this case series, one patient needed re-operation and biliary reconstruction by bile duct stones centered on a clip and Mirizzi syndrome 3 years after the initial operation. In addition, this case was one of four cases of partial cholecystectomy.

Early surgery may be increased by evaluating surgical difficulty with a new diagnostic procedure^1–3^, but a cautious attitude to reduce complications is most important.

However, the risk of bile duct injury has been found to be extremely low in mild cholecystitis, and laparoscopic cholecystectomy could be performed in all cases^20,21^. In recent years, surgical devices and skill’s advance become noticeable. Moreover, laparoscopic surgery has enlarged vision effects, so these effects considered to be advantage to work in acute surgery. In this case series, we surgeons were initially overly cautious about performing laparoscopic surgery with patient who has cardiovascular comorbidities, the rate of early operation is still less than 40% as a result. The reason is not technical anxiety, but concern for the worsening of cardiovascular disease. It is strongly associated with balancing the risk of postoperative bleeding and thrombosis, whether the patient should be interrupted antithrombotic therapy or used bridging anticoagulant therapy during invasive procedures. In fact, we have chosen to discontinue anticoagulants or antiplatelet drugs and heparinized due to concerns about worsening of the comorbidities and fear of perioperative hemorrhage. On the other hand, it is known that discontinuation of medication is a risk of stroke in patients who have been receiving antithrombotic therapy for a long time^22^. However, on patients receiving procedures associated with a lower risk of bleeding, antithrombotic therapy can be continued safely^23^. Previous reports from Germany say that the use of low weight molecule heparin increases postoperative bleeding after laparoscopic cholecystectomy^24^, but subsequently perioperative bleeding and thrombotic complications did not increase in laparoscopic gastroenterological surgery in patients receiving antithrombotic therapy^25,26^. In addition, there have been several literatures that early laparoscopic cholecystectomy in emergencies can be performed safely even under anticoagulant therapy and antiplatelet therapy^27–30^.

## Conclusion

On mild acute cholecystitis up to Grade II, early surgery should be performed regardless of whether there is cardiovascular disease or anticoagulant therapy. If surgeons want to use the bed efficiently, they should not hesitate to perform early surgery to secure hospital beds, especially in the recent covid19 pandemic. These contents could be described in the next revision of the guidelines, and a large-scale prospective study should be planned.

## Data Availability

The datasets analyzed during the current study available from the corresponding author on reasonable request.

## Abbreviations

TG18: Updated Tokyo Guidelines for acute cholangitis and acute cholecystitis
CT: Computed tomography
MRCP: Magnetic resonance cholangiopancreatograph**y**
Pt-INR: International normalized ratio ofprothrombin time
HT: Hypertension
OMI: Old myocardial infarction
DVT: Deep vein thrombosis
AAA: Abdominal aortic aneurysm
ASO: Arteriosclerosis obliterans
Af: Atrial fibrillation
